# A conserved Polϵ binding module in Ctf18-RFC is required for S-phase checkpoint activation downstream of Mec1

**DOI:** 10.1093/nar/gkv799

**Published:** 2015-10-10

**Authors:** Luis J. García-Rodríguez, Giacomo De Piccoli, Vanessa Marchesi, Richard C. Jones, Ricky D. Edmondson, Karim Labib

**Affiliations:** 1 Cancer Research UK Manchester Institute, University of Manchester, Wilmslow Road, Manchester M20 4BX, UK; 2 Division of Biomedical Cell Biology, Warwick Medical School, University of Warwick, Coventry CV4 7AL, UK; 3 MS Bioworks, 3950 Varsity Drive, Ann Arbor, MI 48108, USA; 4 Myeloma Institute for Research and Therapy, University of Arkansas for Medical Sciences, 4301 W Markham #776, Little Rock, AR 72205, USA; 5 MRC Protein Phosphorylation and Ubiquitylation Unit, Sir James Black Centre, College of Life Sciences, University of Dundee, Dow Street, Dundee DD1 5EH, UK

## Abstract

Defects during chromosome replication in eukaryotes activate a signaling pathway called the S-phase checkpoint, which produces a multifaceted response that preserves genome integrity at stalled DNA replication forks. Work with budding yeast showed that the ‘alternative clamp loader’ known as Ctf18-RFC acts by an unknown mechanism to activate the checkpoint kinase Rad53, which then mediates much of the checkpoint response. Here we show that budding yeast Ctf18-RFC associates with DNA polymerase epsilon, via an evolutionarily conserved ‘Pol ϵ binding module’ in Ctf18-RFC that is produced by interaction of the carboxyl terminus of Ctf18 with the Ctf8 and Dcc1 subunits. Mutations at the end of Ctf18 disrupt the integrity of the Pol ϵ binding module and block the S-phase checkpoint pathway, downstream of the Mec1 kinase that is the budding yeast orthologue of mammalian ATR. Similar defects in checkpoint activation are produced by mutations that displace Pol ϵ from the replisome. These findings indicate that the association of Ctf18-RFC with Pol ϵ at defective replication forks is a key step in activation of the S-phase checkpoint.

## INTRODUCTION

Chromosome replication poses a major threat to genome integrity. This can be due to mutations that result from errors during DNA synthesis, or genome rearrangements that originate from defective DNA replication forks, driven by the exposure of single-strand DNA or by the unprotected ends of nascent DNA molecules. For these reasons, defects in chromosome replication play an important role in the early development of human cancer (1), and eukaryotic cells have evolved many adaptive mechanisms that help to preserve genome integrity during the process of DNA replication.

One of the best characterized of these pathways is the S-phase checkpoint response (2–5), which is activated by an increased exposure of single-strand DNA at replication forks, resulting from the combination of defects in DNA synthesis and the ongoing action of the DNA helicase that is responsible for fork progression. The ATR (ATR = ATM related) checkpoint kinase (6) is recruited to areas of increased single-strand DNA (7), by interaction of its regulatory subunit with the single-strand DNA binding protein known as RPA (RPA = Replication Protein A). The recruitment of other checkpoint proteins to the same sites then leads to local activation of ATR and the phosphorylation of a range of downstream targets (5). One of the most important consequences of ATR activation at defective replication forks is the recruitment and activation of a downstream checkpoint kinase called Chk1 in higher eukaryotes or Rad53 (Rad = radiation sensitive) in budding yeast. The downstream kinase then diffuses away from the defective replication forks and induces a wide variety of cellular responses that help to maintain fork stability and thus preserve genome integrity (2–5). Amongst others, these responses include the inhibition of late-firing origins of DNA replication (8,9), the transcription of replication and repair factors (10,11), and the stimulation of dNTP production by regulation of ribonucleotide reductase (12–16).

Activation of the downstream checkpoint kinase at defective replication forks is driven by ATR but also requires an ‘adaptor’ known as Claspin or Mrc1 (Mrc = Mediator of the Replication Checkpoint), which associates with the replisome at replication forks via factors around the DNA helicase and by interaction with the leading strand DNA polymerase ϵ (17–22). Claspin/Mrc1 is phosphorylated by ATR and serves as a scaffold that recruits the downstream checkpoint kinase, promoting activation of the latter by auto-phosphorylation (23–25).

More enigmatically, work with budding yeast has shown that another conserved replication factor called Ctf18-RFC (Ctf = Chromosome Transmission Fidelity; RFC = Replication Factor C) is also needed for activation of the Rad53 downstream checkpoint kinase at defective replication forks (26–29). Ctf18-RFC is one of four ‘clamp loader complexes’, which each contain five related ATPases that serve to load ring-shaped ‘clamps’ around the junctions of primers with template DNA at replication forks (30). All forms of RFC share a common core comprising Rfc2–4, but each also contains a unique largest subunit that confers specificity of action. Rfc1-RFC is essential to load the trimeric Pol30/PCNA clamp around the junction of primer and template DNA at replication forks, where it serves as a processivity factor for DNA polymerases (30). In contrast, Elg1-RFC (Elg = Enhanced Level of Genome Instability) is thought to unload PCNA from nascent DNA after the passage of replication forks (31,32), whereas Rad24-RFC helps to activate the DNA damage checkpoint by loading a trimeric ‘checkpoint clamp’ at sites of damaged DNA (33,34). The action of Ctf18-RFC as a clamp loader is still understood poorly; it has been shown like Rfc1-RFC to be important *in vivo* for efficient association of PCNA with replicating chromatin (27,35), but *in vitro* Ctf18-RFC was found to serve principally as an unloader for PCNA (36). At present, the molecular mechanism by which Ctf18-RFC mediates activation of the DNA replication checkpoint is not understood.

In addition to being required for activation of the S-phase checkpoint, Ctf18-RFC is important during chromosome replication for the establishment of cohesion between sister chromatids (37,38). This is likely to involve the PCNA-dependent recruitment to replication forks of the Eco1 acetyltransferase (Eco = Establishment of cohesion), which acetylates the Smc3 subunit of the cohesion complex and thus counteracts the ‘anti-establishment’ activity of Rad61/Wpl1, which otherwise would destabilise the cohesin ring that encircles pairs of sister chromatids (39). Ctf18-RFC is also required by an unknown mechanism for correct positioning of telomeric chromatin at the nuclear periphery (40).

Uniquely amongst the four clamp loader complexes, all the known roles of Ctf18-RFC also require two additional subunits of the complex called Ctf8 and Dcc1 (Dcc = Defective in sister chromatid cohesion). These are unrelated to Rfc1–5/Elg1/Rad24/Ctf18 and form a heterodimer that associates with Ctf18 (37). Until now, the only clue to the molecular role of Ctf8-Dcc1 in any species came from a study of the human Ctf18-RFC complex, which was found to interact with DNA Pol ϵ that synthesizes the leading strand at DNA replication forks (41). Association of human Ctf18-RFC with Pol ϵ requires not only Ctf8-Dcc1 but also Ctf18, which together form a Pol ϵ-binding module (41). However, the significance of this Pol ϵ-binding module for Ctf18-RFC function in human cells still remains to be explored. Moreover, it was unclear until now whether the association of human Ctf18-RFC with Pol ϵ represented an evolutionarily conserved feature in other species. Here we report that budding yeast Ctf18-RFC associates with Pol ϵ in a very similar manner to the interaction of the human proteins. Importantly, we show that activation of the S-phase checkpoint in budding yeast, downstream of the Mec1 checkpoint kinase, is not only dependent upon the Pol ϵ-binding module of Ctf18-RFC, but also requires the incorporation of Pol ϵ into the replisome. These findings indicate that Pol ϵ serves as a hub for S-phase checkpoint signaling at defective DNA replication forks.

## MATERIALS AND METHODS

### Yeast strains and growth

Supplementary Table S1 lists the *Saccharomyces cerevisiae* strains that were used in this study. Cultures were grown in rich media (YPD) that contained yeast extract (1%), peptone (2%) and glucose (2%). When required, cells were synchronized in G1 by addition of 7.5 μg/ml alpha-factor mating pheromone and released into S phase by washing twice with fresh YPD media. To inhibit ribonucleotide reductase and slow progression through S-phase, hydroxyurea (HU; Sigma-Aldrich H8627) was added to a final concentration of 150mM in solid medium or 200mM in liquid cultures. Cells were arrested in G2-phase by addition of 5 μg/ml nocodazole (Sigma-Aldrich M1404) to the culture medium for one generation time.

For transformation and selection, synthetic complete dropout medium (SC-media) was used with the required supplements. For selection of ura- cells, 5-Fluoroorotic acid (5-FOA; F5001, Melford Laboratories) was added to a final concentration of 1% in SC medium supplemented with uracil.

To make the *ctf18–2A* allele with the *W736A W740A* mutations, the *URA3* cassette was introduced into one copy of the *CTF18* locus in a diploid strain. We then transformed the resultant diploid with a polymerase chain reaction (PCR) product corresponding to the *ctf18–2A* allele followed by the *Kluveromyces lactis TRP1* marker (*K.l.TRP1*), with homology at both ends of the PCR product to genomic *CTF18* sequences either side of the inserted *URA3* gene. Transformants expressing the *K.l.TRP1* marker gene were selected and checked for loss of the *URA3* gene using SC plates supplemented with 5-FOA. 5-FOA resistant clones were sequenced to confirm the formation of the new *ctf18–2A* locus. From the resulting heterozygous diploid strains we generated by tetrad analysis the final haploid *ctf18–*2A strain in which the *CTF18* locus was identical to control cells, except for presence of the *W736A* and *W740A* mutations, and the *K.l.TRP1* marker inserted at the 3′ end of *ctf18–2A*. In parallel, we made an equivalent control strain with the *K.l.TRP1* marker inserted at the 3′ end of wild type *CTF18*.

### Plasmids

Appendix Supplementary Table S2 lists plasmids that were used in this study. Two-hybrid plasmids were made by recombination in budding yeast, by co-transforming digested versions of pGADT7 (Gal4-activation domain-HA tag; Clontech) or pGBKT7 (Gal4-DNA binding domain-MYC tag; Clontech) into yeast cells, together with PCR products that contained the test sequence flanked by 50 bp homology to the digested vector. Subsequently the correctly recombined plasmids were recovered from yeast and retransformed in order to confirm the resulting phenotypes.

### Yeast two-hybrid assays

Two-Hybrid analysis based on the Gal4 transcription factor was performed by co-transformation of derivatives of pGADT7 (Gal4 activation domain; *LEU2* marker) and pGBKT7 (Gal4 DNA binding domain; *TRP1* marker) into the yeast strains PJ69–4A (wild type two-hybrid strain), YLG60 (*ctf8Δ* version of PJ69–4A) or YLG63 (*dcc1Δ* version of PJ69–4A). For each assay, five independent transformed colonies were mixed together in PBS medium and used to make serial dilutions, before spotting 10-fold dilutions from 50 000 to 50 cells onto SC medium lacking tryptophan and leucine (selective for pGADT7 and pGBKT7, but non-selective for the two-hybrid interaction) or SC medium lacking tryptophan, leucine and histidine (selective for the two-hybrid interaction).

### Immunoprecipitation and immunoblotting of proteins from yeast cell extracts

Cells extracts were obtained from 250 ml culture samples (about 2.5 × 10^9^ cells) in the presence of 100 mM potassium acetate (or 50 mM potassium acetate for the experiment in Figure 3D), as previously described (42,43), using a SPEX SamplePrep 6850 Freezer/Mill. For the digestion of chromosomal DNA, extracts were incubated for 30 min at 4°C with 800 units of benzonase (71206–3, Merck Biosciences). We isolated tagged proteins by immunoprecipitation using magnetic Dyna- beads M-270 Epoxy (Invitrogen) coupled to rabbit IgG (Sigma S-1265) or M2 anti-FLAG monoclonal antibody (Sigma F3165). We analysed samples by SDS-PAGE and typically loaded 4 μl of cell extracts and 12 μl of IP samples.

The TAP tag was detected using Peroxidase:Anti-Peroxidase complex (Sigma P-2026). Other proteins were detected by immunoblotting using polyclonal antibodies previously described (18), polyclonal anti-FLAG antibody (Sigma F-7425), 9E10 anti-MYC antibody (Cancer Research UK), polyclonal anti-Rad53 antibody (Santa Cruz sc-6749) and polyclonal antibody specific for a histone H2A peptide containing phosphorylated Serine 129 (Abcam ab15083). Rad53 and γ-H2A immunoblotting was performed with protein samples obtained by trichloroacetic acid precipitation (44). For the experiment in Figure 5A, the signals for hyperphosphorylated and hypophosphorylated Rad53 were quantified using ‘ImageJ’ software.

### Purification of protein complexes and analysis by mass spectrometry

TAP-tagged proteins were purified from 4 l cultures, as described previously (18). For mass spectrometry analysis of protein content, each sample was run in a gel lane that was then cut into 60 bands (Supplementary Figure S1A), or else run for about 2 cm in a gel lane that was cut into 10 bands (Supplementary Figure S1B), before in-gel digestion of proteins with trypsin. The digested peptides were then analysed by nano-liquid chromatography tandem mass spectrometry (MS Bioworks) with an Orbitrap Velos (Thermo Fisher Scientific). Product ion data were searched against the Saccharomyces Genome Database (SGD; www.yeastgenome.org), using the Mascot search engine v2.0.04 (Matrix Science, London, UK) via Mascot Daemon v.2.0.0 (Supplementary Figure S1A).

### Cohesion assays

Cohesion analyses were performed using strains expressing the Tet-repressor fused to GFP and an array of the Tet-operator sites at the *ura3* locus. Cells were arrested in the G2-M phase by the addition of nocodazole and fixed with 8% formaldehyde. We used a Zeiss Axiovert 200M microscope and a Cool Snap HQ camera (Photometrics), controlled via Metamorph acquisition software (Molecular Devices).

## RESULTS

### The association of Ctf18-RFC with DNA polymerase epsilon is conserved from humans to yeast

**Figure 1.**
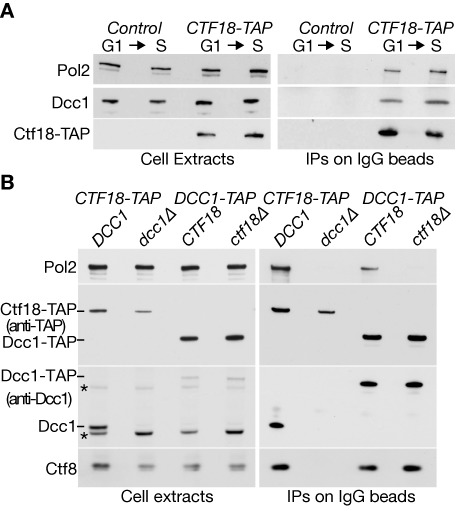
Ctf18 and Ctf8-Dcc1 are required for budding yeast Ctf18-RFC to associate with DNA polymerase epsilon. (**A**) Control cells (YLG98) and *CTF18-TAP* (YLG100) were synchronized at 24°C in the G1-phase of the cell cycle by addition of mating pheromone, before release into S phase for 30 min. Cell extracts were incubated with magnetic beads coupled to rabbit IgG, and the immunoprecipitated proteins were then monitored by immunoblotting. (**B**) Asynchronous cultures of *CTF18-TAP* (YGDP1970), *CTF18-TAP dcc1Δ* (YGDP1971), *DCC1-TAP* (YGDP1972) and *DCC1-TAP ctf18Δ* (YGDP1973) were grown at 24°C, and then processed as in (A). Asterisks in the immunoblots denote non-specific bands.

In a systematic analysis of yeast protein complexes by mass spectrometry (45), Pol2 and Dpb2 (the largest two subunits of Pol ϵ; Dpb = DNA polymerase B subunit 2) were found to co-purify with TAP-tagged Ctf18, but Ctf18-RFC was not observed in purifications of Dpb2-TAP or Dpb3-TAP. We repeated these purifications, and not only detected peptides of Pol2 in samples of purified Ctf18-TAP (Supplementary Figure S1A), but also found that peptides from Ctf18-Ctf8-Dcc1 were specifically enriched in samples of purified Dpb2-TAP (Supplementary Figure S1B). We confirmed these interactions by immunoblotting after isolation of Ctf18-TAP, and found that Ctf18-RFC can associate with Pol ϵ not only in extracts of S-phase cells, but also during G1-phase (Figure 1A).

Ctf18 did not co-purify with Pol2 in the absence of the Dcc1 subunit of Ctf18-RFC (Figure 1B, *CTF18-TAP dcc1Δ*). Similarly, the Dcc1-Ctf8 heterodimer only co-purified with Pol2 in the presence of Ctf18 (Figure 1B, compare *DCC1-TAP* and *DCC1-TAP ctf18Δ*). It thus appears that Ctf18 and Ctf8-Dcc1 are jointly required for Ctf18-RFC to associate with Pol ϵ in budding yeast, mirroring the interaction of the human proteins.

**Figure 2.**
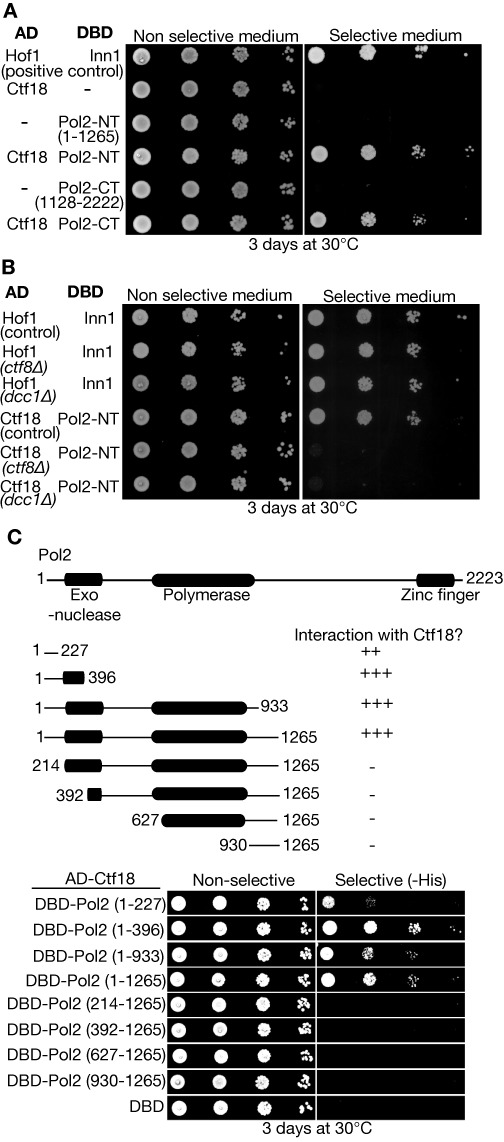
Ctf18-RFC associates with an amino terminal region of Pol2 anterior to the DNA polymerase domain. (**A**) Serial dilutions of yeast cells transformed with the indicated plasmids (AD = protein fused to Gal4-activation domain; DBD = protein fused to Gal4 DNA binding domain) were plated on the indicated media, as described in Materials and Methods. The interaction of Inn1 and Hof1 was used as a positive control for the yeast two-hybrid assay. (**B**) The indicated fusion proteins were tested in the wild type two-hybrid strain (control, PJ69–4A), or in congenic strains lacking Ctf8 (*ctf8Δ*, YLG60) or Dcc1 (*dcc1Δ*, YLG63). Inn1 and Hof1 still interacted in all strains, but Ctf18 only interacted with Pol2NT in the presence of Ctf8 and Dcc1. (**C**) The indicated truncations of Pol2-NT were tested for their ability to interact with Ctf18 in the two-hybrid assay.

Human Ctf18-RFC was shown previously to bind to the amino terminal half of the catalytic subunit of Pol ϵ (41), which contains both the exonuclease and DNA polymerase domains. Using the yeast two-hybrid assay, we found that budding yeast Ctf18 interacted with the amino terminal half of Pol2 (Figure 2A, Pol2NT = Pol2 1–1265), but did not interact with the Dpb2–3–4 subunits of Pol ϵ (Supplementary Figure S2; data not shown for Dpb2). Ctf18 also interacted in the same assay with the carboxy terminal half of Pol2 (Figure 2A, Pol2CT = 1128–2222), potentially indicating a second binding site for Ctf18 within Pol ϵ. However, Pol2CT interacts with itself in the two-hybrid system (Supplementary Figure S3), and we thus cannot exclude that endogenous Pol2 serves as a bridge between Pol2CT and Ctf18 in this assay (with Ctf18 binding the amino terminal domain of endogenous Pol2, and Pol2CT binding to the carboxy-terminal half of endogenous Pol2).

The interaction of Ctf18 with Pol2NT required both Ctf8 and Dcc1 (Figure 2B), indicating that the assay reflected the specific association of Ctf18-RFC with Pol ϵ as described above. Analysis of truncated versions of Pol2NT indicated that the interaction with yeast Ctf18-RFC was mediated by a small region at the amino terminus of Pol2, overlapping partially with the exonuclease but distinct from the DNA polymerase domain (Figure 2C).

### Mutations in the extreme carboxyl terminus of Ctf18 prevent association with Ctf8-Dcc1 and DNA polymerase epsilon

**Figure 3.**
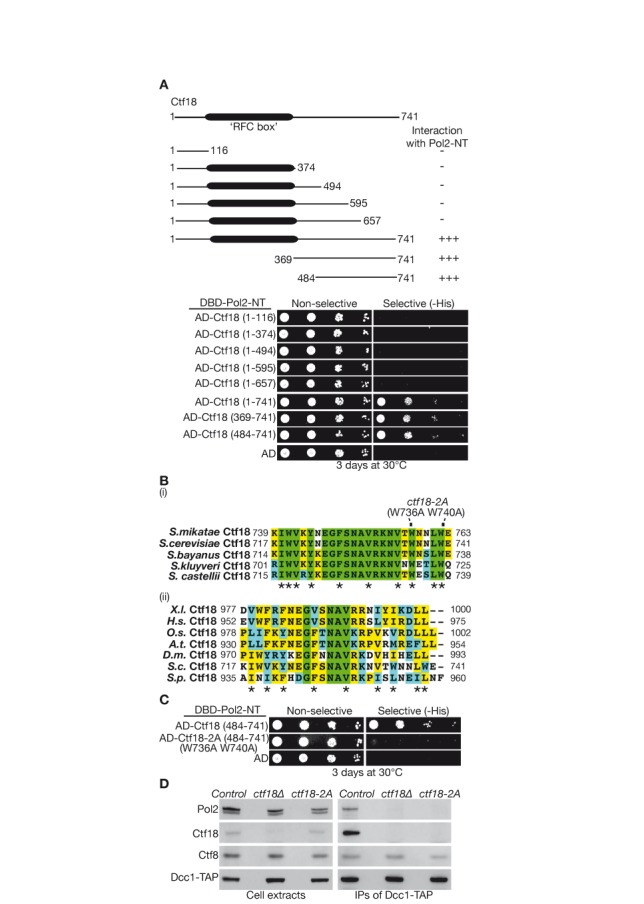
Mutations in a conserved motif at the carboxyl terminus of Ctf18 disrupt the interaction of budding yeast Ctf18-RFC with DNA polymerase epsilon. (**A**) The indicated truncations of Ctf18 were tested for their ability to interact with Pol2-NT in the two-hybrid assay. (**B**) (i) Alignment of the carboxyl terminus of Ctf18 from each of the indicated budding yeast species, generated with ClustalW and BOXSHADE software. Asterisks denote 10 conserved hydrophobic residues within this region. (ii) An analogous alignment of the end of the Ctf18 protein from diverse eukaryotic species, showing conservation of 9/10 hydrophobic residues (X.l. = *Xenopus laevis*; H.s = *Homo sapiens*; O.s. = *Oryza sativa*; A.t. = *Arabidopsis thaliana*; D.m. = *Drosophila melanogaster*; S.c. = *Saccharomyces cerevisiae*; S.p. = *Schizosaccharomyces pombe*). (**C**) The *W736A W740A* mutations prevent interaction of yeast Ctf18 484–741 with Pol2NT in the yeast two-hybrid assay. (**D**) Asynchronous cultures of *DCC1-TAP* (*Control*, YLG301), *DCC1-TAP ctf18Δ* (*ctf18Δ*, YVM850) and *DCC1-TAP ctf18–2A* (*ctf18–2A*, YLG303) were grown at 24°C, and then processed as in Figure 1A.

Truncations of yeast Ctf18 implicated the unique carboxy terminal half of the protein, beyond the ‘RFC box’ that is shared with other large subunits of RFC complexes, as being required for interaction with Pol ϵ (Figure 3A). Alignment of this region of Ctf18 in orthologues from closely related budding yeast species showed that the final 25 amino acids of Ctf18 are particularly well conserved, and contain 10 invariant hydrophobic residues (Supplementary Figure S4A), which might contribute to protein–protein interactions. This region of Ctf18 is also well conserved in orthologues of Ctf18 from more distantly related eukaryotic species such as humans, including nine of the 10 hydrophobic residues from the C-terminal tail of yeast Ctf18 (Supplementary Figure S4B). Notably, the C-terminal 23 residues of human Ctf18 were found to be sufficient *in vitro* to produce a ternary complex with Ctf8-Dcc1 and Pol ϵ (41).

To try and identify an allele of yeast *CTF18* that would specifically disrupt the putative Pol-epsilon interaction module, allowing us to investigate its functional significance for the first time in any species, we mutated each of the last 25 amino acids of Ctf18 (Figure 3B) to alanine, except for A729 that we mutated to glycine or threonine. Using the two-hybrid assay, we found that none of the single mutations abolished the interaction of Ctf18 with Pol2NT or Dcc1, but mild defects in both interactions were produced by mutation of either W740 or E741 at the extreme carboxyl terminus of Ctf18 (Supplementary Figure S5). As aromatic residues can be particularly important in mediating protein–protein interactions, we combined mutation of W740 with mutation of the neighbouring aromatic residue W736 (Figure 3B), and found that Ctf18 484–741 with the W736A W740A mutations was unable to interact with Pol2NT in the two-hybrid assay (Figure 3C). These data indicate that the carboxyl terminus of Ctf18 contains a Pol ϵ interaction motif that has been conserved from yeast to humans.

The W736A W740A mutations were then introduced into the endogenous *CTF18* locus of budding yeast cells to create the *ctf18–2A* allele (see Materials and Methods). The Dcc1-Ctf8 heterodimer was unable to interact with Ctf18–2A or with Pol ϵ in *ctf18–2A* cells (Figure 3D, IPs of Dcc1-TAP), confirming that the W736A W740A mutations in Ctf18 disrupted the Pol-epsilon binding module of Ctf18-RFC, despite the Ctf18–2A protein being expressed to a similar level as wild type Ctf18 (Figure 3D, Cell extracts).

### Integrity of the Pol ϵ binding module of Ctf18-RFC is required downstream of the Mec1 protein kinase for activation of the S-phase checkpoint

**Figure 4.**
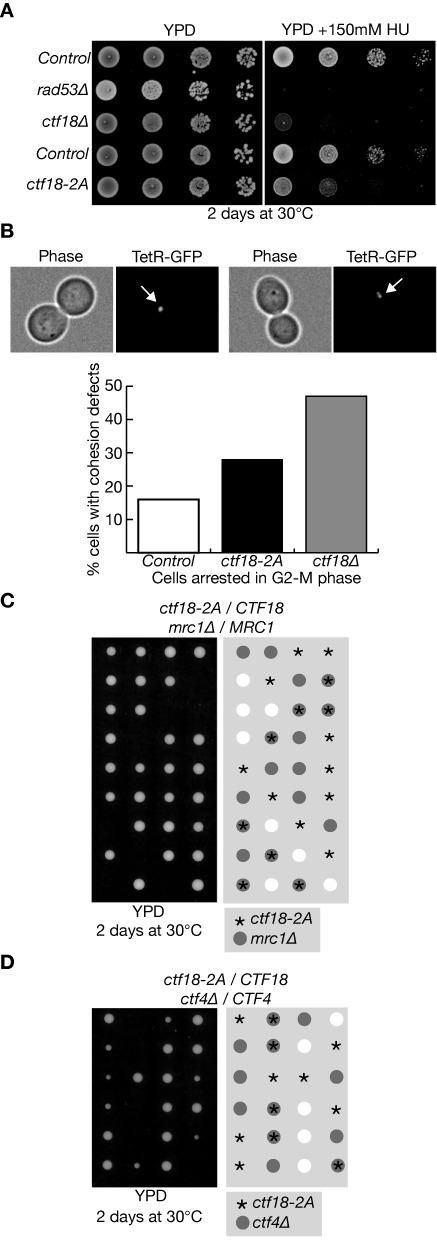
Phenotypes of the *ctf18–2A* allele. (**A**) Serial dilutions of control cells (W303–1a), *rad53Δ* (YAC53), *ctf18Δ* (YVM164), *CTF18::K.l.TRP1* (wild type *CTF18* but with the *K.l.TRP1* marker inserted into the genomic locus, after the STOP codon of *CTF18*; YLG316) and *ctf18–2A* (also with the *K.l.TRP1* marker inserted after the STOP codon of *CTF18*; YLG320) were plated on YPD medium, or YPD supplemented with 150mM hydroxyurea (HU), then grown for 2 days at 30°C. (**B**) Control cells (*CTF18-K.l.TRP1;* YLG445), *ctf18–2A (ctf18–2A K.l.TRP1*; YLG447) and *ctf18Δ::K.l.TRP1* (YLG449), all expressing the Tet-repressor fused to GFP and with an array of the Tet-operator sites at the *ura3* locus, were grown at 30°C and then arrested in G2-M phase by addition of nocodazole. Defects in sister chromatid cohesion were scored microscopically, by examining 100 cells and determining the percentage with two dots of TetR-GFP instead of one (examples of each class are shown in the upper panels). (**C**) Diploid cells with the genotype *ctf18–2A / CTF18 mrc1Δ / MRC1* (YLG292) were sporulated and then subjected to tetrad analysis on YPD medium. The image was taken after two days growth at 30°C. (**D**) Tetrad analysis of the meiotic progeny of *ctf18–2A / CTF18 ctf4Δ / CTF4* (YLG263).

Cells lacking Ctf18 are highly sensitive to DNA replication stress induced by treatment the ribonucleotide reductase inhibitor hydroxyurea, and have a strong defect in the establishment of cohesion between sister chromatids during chromosome replication (28,37). *ctf18–2A* cells are also sensitive to growth in the presence of hydroxyurea (Figure 4A and Supplementary Figure S6) and are defective in sister chromatid cohesion (Figure 4B), but both defects are less severe than those observed in the complete absence of Ctf18. This might indicate that Ctf18–2A is partially defective in the various functions of wild type Ctf18, or that the mutated Ctf18–2A protein is specifically defective in a subset of functions. We thus tested whether *ctf18–2A* shared other reported phenotypes of *ctf18Δ* cells, by crossing *ctf18–2A* to *mrc1Δ* or *ctf4Δ*, since the combined absence of Ctf18 and Mrc1, or Ctf18 and Ctf4, has been found previously to cause synthetic lethality (29,46). Strikingly, both *ctf18–2A mrc1Δ* (Figure 4C) and *ctf18–2A ctf4Δ* (Figure 4D) were found to be inviable. This raised the possibility that at least one function of Ctf18-RFC might be lost when the integrity of the Pol-epsilon binding module is disrupted.

**Figure 5.**
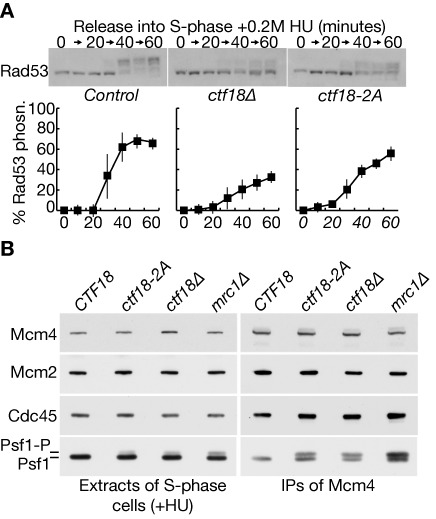
Disruption of the Pol ϵ binding module of Ctf18-RFC blocks activation of the S-phase checkpoint, downstream of Mec1. (**A**) Control cells (W303–1a), *ctf18–2A* (YLG249) and *ctf18Δ* cells (YVM164) were arrested in G1-phase at 24°C and then released into fresh medium containing 0.2M hydroxyurea for the indicated times. Rad53 hyperphosphorylation was monitored by immunoblotting (upper panels), and the data from three such experiments were then quantified (lower panels; ‘% Rad53 phosphorylation’ was calculated from the ratio of hyperphosphorylated to hypophosphorylated Rad53). (**B**) Control cells (YLG426), *ctf18–2A* (YLG423), *ctf18Δ* (YLG421) and *mrc1Δ* (YGDP993) were arrested in G1-phase at 24°C, before release for 90 min into S-phase in the presence of 0.2M hydroxyurea. Mcm4–5FLAG was immunoprecipitated from cell extracts and the indicated proteins monitored by immunoblotting.

Finally, we tested whether the S-phase checkpoint pathway is defective in *ctf18–2A* cells. When *ctf18Δ* or *mrc1Δ* cells enter S-phase in the presence of hydroxyurea, the S-phase checkpoint pathway cannot be activated at the defective replication forks that are established from early replication origins, leading to increased DNA damage and the firing of late replication origins (17,26–27). Activation of the Rad53 checkpoint kinase is defective but is not abolished in *ctf18Δ* or *mrc1Δ* cells under such conditions, since the unprotected replication forks activate a DNA-damage branch of the checkpoint pathway, which is independent of Ctf18 and Mrc1 (26,27). We found that Rad53 activation was defective when *ctf18Δ* or *ctf18–2A* cells entered S-phase in the presence of hydroxyurea (Figure 5A), and this was associated with increased phosphorylation of Serine 129 of histone H2A, which provides a marker for DNA damage (Supplementary Figure S7). These data suggested that activation of the S-phase checkpoint is defective when the Pol ϵ binding module of Ctf18-RFC has been mutated.

To confirm these findings and explore which stage of the S-phase checkpoint pathway is defective in *ctf18–2A* cells, we examined the Mec1-dependent phosphorylation of the CMG helicase (42). When *rad53Δ, mrc1Δ* or *ctf18Δ* cells are treated with hydroxyurea, we found previously that Mec1-signalling at defective replication forks is increased, as reflected by increased Mec1-dependent phosphorylation of the Psf1 subunit of the Cdc45-MCM-GINS (CMG) DNA helicase (42). Importantly, the same effect is observed in hydroxyurea-treated *sld3-A dbf4–4A* cells (42), in which the checkpoint kinases are activated normally but the key Rad53 phosphorylation sites in the initiation factors Sld3 and Dbf4 have been mutated (47), leading to the firing of late origins and an increase in DNA replication stress. This means that accumulation of the CMG helicase with hyper-phosphorylated Psf1 is a sensitive marker for the aberrant firing of late-origins in cells treated with hydroxyurea (42), reflecting a specific failure to activate the S-phase checkpoint response at some point downstream of the Mec1 kinase.

We synchronized wild type cells, *ctf18Δ, mrc1Δ* and *ctf18–2A* in G1-phase at 24°C by treatment with mating pheromone, before release into S-phase for 90 min in the presence of 0.2M hydroxyurea. Critically, the Psf1 subunit of the CMG helicase accumulated in a hyperphosphorylated form in *ctf18–2A* cells as well as in *ctf18Δ* and *mrc1Δ* (Figure 5B). This was particularly evident when the isolated CMG helicase was monitored in the immune-precipitates of Mcm4–5FLAG (Figure 5B, Psf1 in IPs of Mcm4–5FLAG), but could also be detected in the cell extracts (Figure 5B, Psf1 in extracts of S-phase cells +HU), reflecting the greater proportion of total GINS that is present at replication forks when early and late origins fire under conditions of replication stress. These data indicate that the integrity of the Pol-epsilon binding module of Ctf18-RFC is required for activation of the S-phase checkpoint in budding yeast, downstream of Mec1 activation.

### S-phase checkpoint activation requires incorporation of Pol ϵ into the replisome

Previous work indicated that DNA polymerase epsilon is important for activation of the S-phase checkpoint (48–50). However, the mechanism was unclear and it was not known whether incorporation of Pol-epsilon into the replisome was important for checkpoint activation. We and others previously showed that Dpb2 links Pol ϵ to the CMG helicase within the replisome, by interaction of the amino-terminal domain of Dpb2 with GINS, and the remainder of Dpb2 with Pol2 (51). Although depletion of Dpb2 blocks CMG assembly during the initiation of chromosome replication, we found that this could be rescued by over-expression of Dpb2NT, producing a replisome that lacks DNA polymerase epsilon, despite the presence of wild type Pol2 (51). Therefore, we used this experimental system to test directly whether S-phase checkpoint activation requires the incorporation of DNA polymerase epsilon into the replisome.

**Figure 6.**
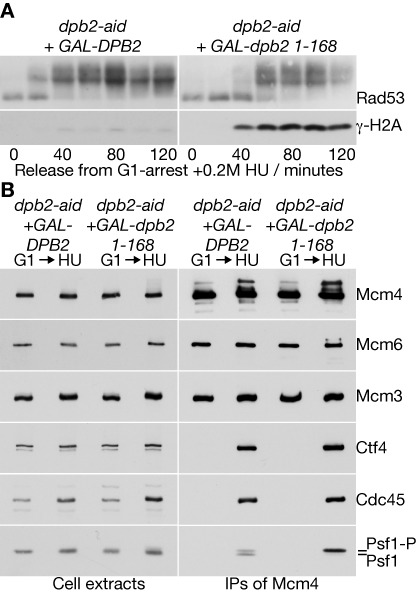
Incorporation of DNA polymerase epsilon into the replisome is required for activation of the S-phase checkpoint, downstream of Mec1. (**A**) Control *dpb2-aid GAL-DPB2* (YCS394) and *dpb2-aid GAL-dpb2 1–168* (YCS396) were arrested in G1-phase at 24°C in YPRaff medium. Cells were then switched for 35 min to YPGal medium containing mating pheromone, before addition of 0.5 mM auxin (indole-3-acetic acid) and incubation for 60 min. Samples were then washed into fresh YPGal medium lacking mating pheromone but containing auxin. Samples were taken at the indicated times, and cell extracts were used to monitor the phosphorylation of Rad53 and Histone H2A by immunoblotting. (**B**) Cells were grown as in (A) and released from into S-phase in the presence of hydroxyurea for 90 min. Mcm4–5FLAG was immunoprecipitated from cell extracts and the indicated proteins monitored by immunoblotting.

Using cells in which the endogenous *DPB2* gene was fused to the auxin-inducible degron (52), we expressed either wild type Dpb2 or the N-terminal fragment comprising Dpb2 1–168 in G1-phase cells and then depleted Dpb2-aid. Upon release into S-phase in the presence of hydroxyurea, checkpoint activation was normal in cells expressing wild type Dpb2, as reflected by the rapid hyper-phosphorylation of Rad53 (Figure 6A, *dpb2-aid GAL-DPB2*). In cells expressing Dpb2 1–168, however, the activation of Rad53 was delayed and occurred concomitantly with the induction of γ-H2A phosphorylation, indicative of DNA damage at unprotected replication forks (Figure 6A, *dpb2-aid GAL-dpb2 1–168*). Immunoprecipitation of the Mcm4 subunit of the CMG helicase indicated that helicase assembly was equally efficient in both strains, consistent with our previous findings (51). Importantly, the Psf1 subunit of CMG was hyper-phosphorylated when cells expressing Dpb2NT were exposed to hydroxyurea (Figure 6B), indicating that incorporation of Pol ϵ into the replisome is required downstream of Mec1, for activation of the S-phase checkpoint in budding yeast.

## DISCUSSION

The Ctf8-Dcc1 heterodimer and the extreme C-terminus of Ctf18 together form a Pol ϵ-binding module in Ctf18-RFC (41), which our data show to have been conserved from yeast to humans. In budding yeast, the Pol ϵ-binding module of Ctf18-RFC is required for activation of the S-phase checkpoint in response to defects in DNA replication (Figure 5). Previous studies indicate that Ctf18-RFC is required for activation of the Rad53 checkpoint kinase at stalled DNA replication forks (26–29), downstream of Mec1 (42). These features of Ctf18-RFC are analogous to the role of Mrc1 in checkpoint activation, and it will be important in future studies to explore how Ctf18-RFC contributes to the recruitment and auto-activation of the Rad53 checkpoint kinase under such conditions.

Our data highlight two important new aspects of S-phase checkpoint activation: incorporation of DNA polymerase epsilon into the replisome and recruitment of Ctf18-RFC to the amino terminus of Pol2, adjacent to the exonuclease domain. These features should position Ctf18-RFC close to the ssDNA that forms an important signal for the checkpoint upon replication stress, and would thus bring Ctf18-RFC into the proximity of the activated Mec1 checkpoint kinase. It is striking that Mrc1 also associates with Pol ϵ (21), although it has not yet been possible to test directly whether the association of Mrc1 with Pol ϵ is required for activation of the S-phase checkpoint. One interesting model for future investigation would be that Pol ϵ serves as a scaffold upon which both Mrc1 and Ctf18-RFC recruit Rad53, facilitating auto-phosphorylation of the latter *in trans*. Other possibilities could also be envisaged at this stage, and ultimately it will be important to try and establish biochemical systems with which to reconstitute Rad53 activation by Mrc1 and Ctf18-RFC.

Since Ctf8 and Dcc1 appear to be required for all the known roles of Ctf18-RFC at DNA replication forks (37,40,53), in addition to activation of the S-phase checkpoint (26,29), it will be interesting in future studies to explore how other functions of Ctf18-RFC might be modulated by association with Pol ϵ. For example, our data indicate that the integrity of the Pol ϵ-binding module of Ctf18-RFC is also important for sister chromatid cohesion (Figure 4B), although the cohesion defect of *ctf18–2A* is less severe than that observed in *ctf18Δ* cells. Recruitment of Ctf18-RFC to the leading strand DNA polymerase might facilitate (without being essential for) the loading of PCNA around the nascent leading strand DNA, which otherwise might receive less PCNA than the nascent lagging strand that is synthesized by a process involving repeated priming and PCNA loading events. Such a mechanism might aid the recruitment of the Eco1 acetyltransferase to both of the nascent sister chromatids, as well as potentially helping to stimulate other PCNA-linked processes such as chromatin assembly on the newly synthesised leading strand DNA (54). It thus seems clear that future studies of Ctf18-RFC will still have much to contribute to our understanding of the some of the most enigmatic features of chromosome replication in eukaryotic cells.

## Supplementary Material

SUPPLEMENTARY DATA
