# Supplementary material for: A conserved Polϵ binding module in Ctf18-RFC is required for S-phase checkpoint activation downstream of Mec1
Source: Nucleic Acids Res. 2015 Oct 10;43(18):8830–8. doi: 10.1093/nar/gkv799 (PMC4605302; doi:10.1093/nar/gkv799)
Supplement: SUPPLEMENTARY DATA [file supp_43_18_8830__index.html]

A conserved Polϵ binding module in Ctf18-RFC is required for S-phase checkpoint activation downstream of Mec1 — SUPPLEMENTARY DATA 

# A conserved Polϵ binding module in Ctf18-RFC is required for S-phase checkpoint activation downstream of Mec1

## SUPPLEMENTARY DATA

- SUPPLEMENTARY DATA
