# Supplementary material for: A conserved Polϵ binding module in Ctf18-RFC is required for S-phase checkpoint activation downstream of Mec1
Source: Nucleic Acids Res. 2015 Oct 10;43(18):8830–8. doi: 10.1093/nar/gkv799 (PMC4605302; doi:10.1093/nar/gkv799)
Supplement: SUPPLEMENTARY DATA [file supp_gkv799_nar-01503-h-2015-File008.pdf]

Legends to Supplementary Figures

Supplementary Figure S1

Ctf18-RFC co-purifies with DNA polymerase epsilon. (**A**) Ctf18-TAP was purified from 4 litres of cell culture, and the final material was resolved in one lane of a 4-12% gradient gel, which was then cut into 60 bands, before analysis of the protein content of each band by mass spectrometry. The histograms show the number of detected peptides per band of the gel, for each of the indicated proteins, with Mascot scores indicated in brackets and bold. (**B**) Summary of mass spectrometry data after two-step purifications (IgG-Sepharose and release of bound material with TEV protease, then Calmodulin-affinity resin) of cell extracts from *DPB2-TAP* and control strains. For each protein detected in the mass spectrometry analysis, the number of spectral counts is shown for each strain, indicating that Ctf18-Dcc1-Ctf8 were specifically enriched upon purification of DNA polymerase epsilon.

Supplementary Figure S2

Ctf18 does not interact with the Dpb3 or Dpb4 subunits of Pol ε in the yeast two-hybrid assay. Two-hybrid analysis was performed as in Figure 2, indicating that Ctf18-RFC did not interact in this assay with Dpb3 or Dpb4 (**A**). As a control, we confirmed that Dpb3 and Dpb4 were able to interact with each other in the same assay (**B**).

Supplementary Figure S3

Pol2CT interacts with itself in the yeast two-hybrid assay. The indicated fragments were tested in the yeast two-hybrid assay, as above in Figure 2A.

Supplementary Figure S4

The Pol ε binding module of Ctf18-RFC comprises a conserved motif at the end of Ctf18 that interacts with Dcc1-Ctf8 and Pol2NT. (**A**) Alignment of the carboxy terminal half of Ctf18 from the indicated budding yeast species, generated as in Figure 3B. Asterisks denote 10 conserved hydrophobic residues at the end of Ctf18. (**B**) An equivalent alignment of the carboxy terminal half of Ctf18 from the indicated species (X.l. = *Xenopus laevis*; H.s = *Homo sapiens*; D.m. = *Drosophila melanogaster*; S.p. = *Schizosaccharomyces pombe*; O.s. = *Oryza sativa*; S.c. = *Saccharomyces cerevisiae*).

Supplementary Figure S5

Residues at the extreme carboxyl terminus of Ctf18 are important for interaction with Pol2NT and Dcc1. The indicated Ctf18 fragments were tested for their ability to interact with Pol2NT (**A**) and Dcc1 (**B**) in the two-hybrid assay, as in Figures 2 and 3.

Supplementary Figure S6

Comparison of the hydroxyurea sensitivity caused by various mutations in Ctf18-RFC. Cells were processed as for Figure 4A.

Supplementary Figure S7

Increased phosphorylation of Serine 129 of histone H2A indicates DNA damage in ctf18-2A upon release into S-phase in the presence of hydroxyurea. One of the replicates from Figure 5A was used to monitor DNA content by flow cytometry (upper panels) and γ-H2A by immunoblotting (lower panels). Ponceau-stained total protein provided a loading control.

Garcia-Rodriguez Fig S1

A

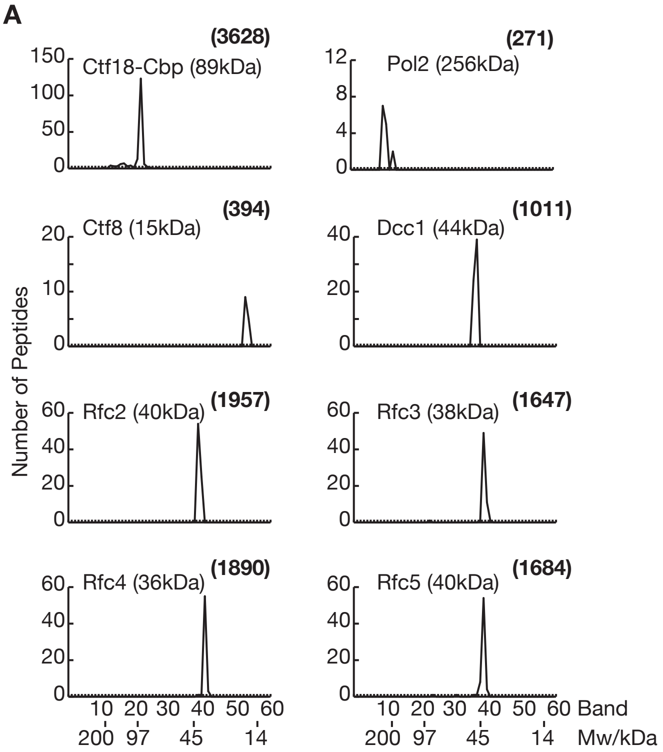

B

**Protein**	**Mw (kDa)**	**Spectral counts (*DPB2-TAP*)**	**Spectral counts (*Control*)**
Dpb2-TAP	99	1193	11
Dpb3	23	157	2
Dpb4	22	218	0
Pol2	256	4286	146
Ctf18	84	91	6
Dcc1	44	19	2
Ctf8	15	25	0

Garcia-Rodriguez Fig S2

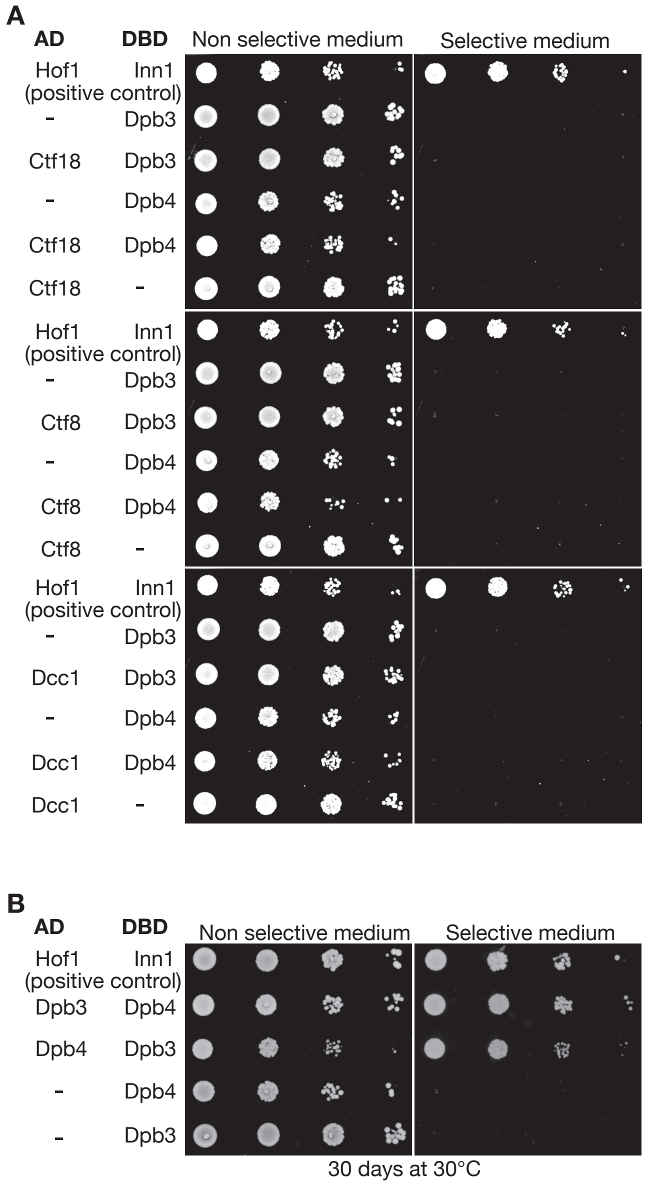

Garcia-Rodriguez Fig S3

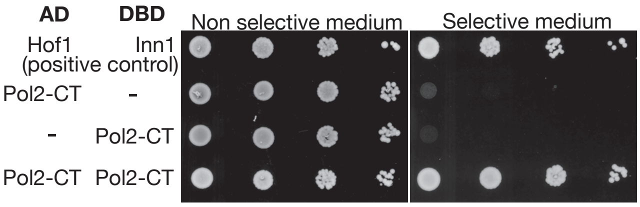

Garcia-Rodriguez Fig S4

A

```
  S. mikatae Ctf18 506 SALVPLVFFQTFGDIANKDDIRMKNSEYERRELRRANLDVVDLIMRHISVQSPLMASFTN
S. cerevisiae Ctf18 484 SALVPLVFFQTFGDIANKDDIRMKNSEYEQRELKRANSDIVSLIMRHISVQSPLMASFTD
  S. bayanus Ctf18 484 SSLVPLVFFQTFGDIANKDDIRMKNSEYEQRELKRANLDIVDSIIRHISVESPLMASFTD
 S. castellii Ctf18 481 CSIVPLAFFQNFGDVANKEDMRIKNAEFENREIQKSNADILSSLIHRISMENPKMMSFID
  S. kluyveri Ctf18 466 SSLVPMAFFQLFSDIANKDDSLIKSVDYEVRESIKHNFSITGSLLDRT---SPRSRVYLN

  S. mikatae Ctf18 566 RKSLIFEILPYLESMISSDFNKIKNLKLKQVIMETLVQLLKSFQLNLIQNRSEIYDARSG
S. cerevisiae Ctf18 544 RKSLIFEILPYLDSMISSDFNKIRNLKLKQAIMEELVQLLKSFQLNLIQNRSEGFDVRGG
  S. bayanus Ctf18 544 RNSLVFQILPYLDSMVSSDLNKVKNLKFKQTIIETLVQVMKNFQLSLMENRSDGFDVRGG
 S. castellii Ctf18 541 RNTLIFDILPYVDRMLSSDISKVKDLTVKSAMLEDLLNILNYFVLKITQVPAFGMEIKNS
  S. kluyveri Ctf18 523 KKNLILEVLPYMDYIITSDLSKIRSMPLRQSMVDNIVPVLKDFGLTLEQRHDP--TSRSM

  S. mikatae Ctf18 626 LVIDPPIEEVVLLNPKHISEVQQKRPNNLSSLLAKIEENKAKKRHIDQVTRDRLQSQEIH
S. cerevisiae Ctf18 604 LTIDPPIDEVVLLNPKHINEVQHKRANNLSSLLAKIEENRAKKRHIDQVTEDRLQSQEMH
  S. bayanus Ctf18 604 LIIDPPIDKVVLLDPKHINKVQHKRPNNLNSLLAKIEENKVKKRHIDQVTKERFQSQEIH
 S. castellii Ctf18 601 LTLDPPIDKVVLLEPSSVKNATFKRPTMLHLLLAKIEERKVKKRRIDKVEKQLQTEEEDR
  S. kluyveri Ctf18 581 LCIQPPLDEVTLLDEKRVKEVATKRPGLLNFLLAKVEECEVKKRTIDKVNRERLENEE--

  S. mikatae Ctf18 686 TKKAKTALTSSSSTIDFFKNQYGLLKQNQESFGTQKST---------VSDEPDQV-DDST
S. cerevisiae Ctf18 664 SKKVKTGLNSSSSTIDFFKNQYGLLKQTQELEETQKTI---------GSDETNQA-DDCN
  S. bayanus Ctf18 664 TKKAKMASNSSSSTIDFFKNQYRIVNQTQEASGGRKNV---------GSSEP----DENT
 S. castellii Ctf18 661 ATKKSKISSGRGSTIDFFKSQYSAINKETASSQDIPKQ---------NADAAGRAGSKNE
  S. kluyveri Ctf18 639 -NRKRSKTSAVSTPVDFFKSQYSSIKTKSSDVSKRASTPELRRSRNFISEGSNTTTATNE

  S. mikatae Ctf18 736 QKIKIWVKYNEGFSNAVRKNVTWNNLWE
S. cerevisiae Ctf18 714 QTVKIWVKYNEGFSNAVRKNVTWNNLWE
  S. bayanus Ctf18 711 EEIKIWVKYKEGFSNAVRKNVTWNSLWE
 S. castellii Ctf18 712 ETTRIWVRYKEGFSNAVRKNVTWESLWQ
  S. kluyveri Ctf18 698 EKIRIWVKYKEGFSNAVRKNVNWETLWQ
                            *** *   *   *   * *  **
```

B

```
X.l. Ctf18 724 LPFLPVAFHLLFAASNVP---RIAYPS-SHYEAQSKLNQMQNLLNAMVSEISPAIRTRVG
H.s. Ctf18 698 PPFLPVAFHVLFASSHTP---RITFPS-SQQEAQNRMSQMRNLIQTLVSGIAPATRSRAT
D.m. Ctf18 735 LQYGFVAWHLLFATLAWP---KIAFPT-RGFEFQQKSTNQRNIFQALCKGVTTSALGVGQ
S.p. Ctf18 718 IPYSIIHFHYLYATPEKC---RLPHPPRSDLEALKLYRTRKEILDSFISTLNAYENQMHG
O.s. Ctf18 727 QPPVAITISQLVAQVEKP---NIEWPK-SLQRCRTMLLEKKDKLKTWQNQMSPLISRHLS
S.c. Ctf18 484 SALVPLVFFQTFGDIANKDDIRMKNSEYEQRELKRANSDIVSLIMRHISVQSPLMASFTD

X.l. Ctf18 780 PQSLVLDALCLLLDVLSP-KLRPVNTQLFSTKEKQQLAELINTMLAYNLTYHQERTMEGQ
H.s. Ctf18 754 PQALLLDALCLLLDILAP-KLRPVSTQLYSTREKQQLASLVGTMLAYSLTYRQERTPDGQ
D.m. Ctf18 791 GGTLLLDTVPLLKRILSP-QLRSVAVQLLSPKEQQDLRHTIEVMVDLGLTFVQVKSQEGH
S.p. Ctf18 775 ERSILLELIRTILITINP-TLKQKEDSMPRSALPSKIEHAINILNHYNLRFQQLPVGDGN
O.s. Ctf18 783 VESFVEDIASPFLHIISPSSLRPVALNLLSEREKDELVQLVDTMVSYSITYRNTKLEPQE
S.c. Ctf18 544 RKSLIFEILPYLDSMISS-DFNKIRN---LKLKQAIMEELVQLLKSFQLNLIQNRSEGFD

X.l. Ctf18 839 YVYK--LDPNVEDVCRFP--DLPVRKPLTYQTKQLIAREIELERMRRTEAFQQARNAGRD
H.s. Ctf18 813 YIYR--LEPNVEELCRFP--ELPARKPLTYQTKQLIAREIEVEKMRRAEASARVENSPQV
D.m. Ctf18 850 YVFQ--TEPDLDALSAFP--GYTG-LSLPYFSRQLIAREVDLERIRRAAPKGGAPSAPAA
S.p. Ctf18 834 YVYR--LEPPLDELVWDA--PTSS-----YSVRQMLSQELLKKRLADIKKQTLTDNPTSS
O.s. Ctf18 843 RISGSMVSPDVPSLSLDP--AINDIINFKVE-KQKIMKDSAGKLLNQANEGDKRNEVSVS
S.c. Ctf18 600 VRGGLTIDPPIDEVVLLNPKHINEVQHKRANNLSSLLAKIEENRAKKRHIDQVTEDRLQS

X.l. Ctf18 895 NTTAAAAVKTADPKGAKSAAKPAALNHEQRLENIMKKATFEEKPEKDFFG-RQIVKKVAA
H.s. Ctf18 869 DGSPPGLEGLLGGIGEKGVHRPAPRNHEQRLEHIMRRAAREEQPEKDFFG-RVVVRSTAV
D.m. Ctf18 905 KKKTSGAAAQLPNHLQTLKPKPISASNMH--------SAPKQQLTKDFFG-RITHKSTST
S.p. Ctf18 885 NSSRKRKDFNSG-----------------------------KAIKRDFFG-RIISEPKSE
O.s. Ctf18 900 EKKSALVSTKSTSKSNPTTLKMQLSSASSMSGKDPAPAKKHSNHGINFFD-RFRKERPVD
S.c. Ctf18 660 QEMHSKKVKTG---------------------------LNSSSSTIDFFKNQYGLLKQTQ

X.l. Ctf18 954 PVT-ASANQEESVERRIGKAVGNSDVWFRFNEGVSNAVRRNIYIKDLL--
H.s. Ctf18 928 LSAGDTAPEQDSVERRMGTAVGRSEVWFRFNEGVSNAVRRSLYIRDLL--
D.m. Ctf18 956 -----------NSAEESKTDAIVKSPIWYRYKEGFNNAVRKDVHIHELL--
S.p. Ctf18 915 AVT----SNNAALNTGDHPVSIKHAINIKFHDGFSNAVRKPISLNEILNF
O.s. Ctf18 959 AKA------RNDAGQQVATTLRDSRPLIFKYNEGFTNAVKRPVKVRDLLL-
S.c. Ctf18 693 ELEETQKTIGSDETNQADDCNQTVKIWVKYNEGFSNAVRKNVTWNNLWE-
                                   *  * *   *   *   * *  **
```

Garcia-Rodriguez Fig S5

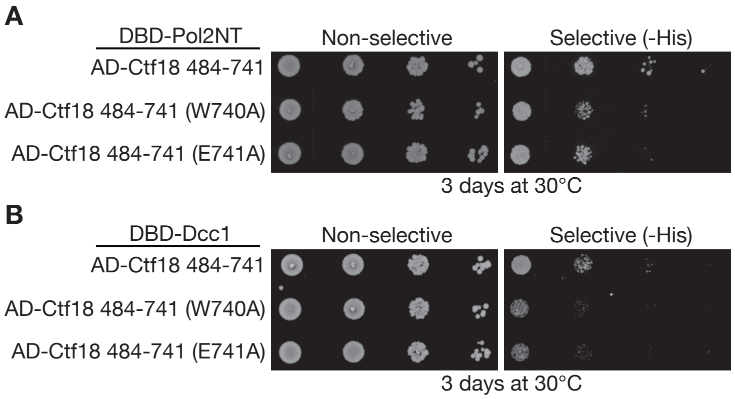

Garcia-Rodriguez Figure S6

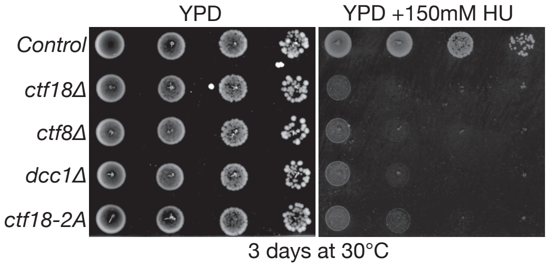

Garcia-Rodriguez Figure S7

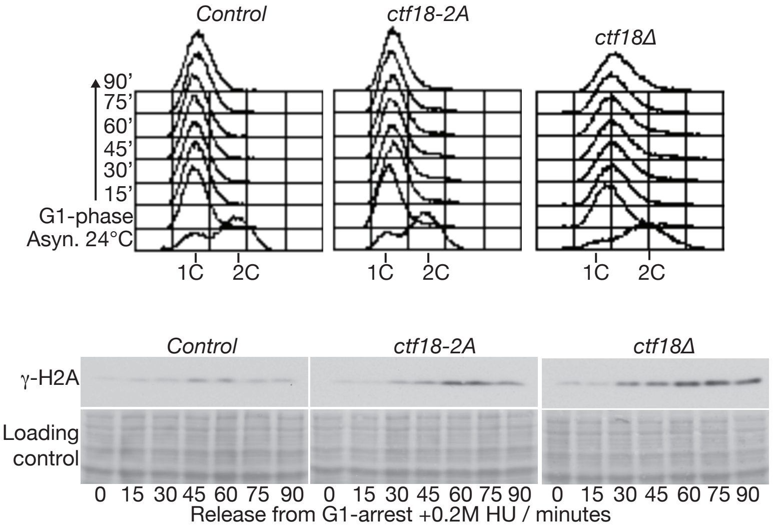

Strain	Genotype	Source
W303-1a	*MAT***a** *ade2-1 ura3-1 his3-1 trp1-1 leu2-3, 112 can1-100*	R. Rothstein
YAC53	*MAT***a** *sml1Δ::HIS3 rad53Δ::ADE2*	This study
YASD375	*MAT***a** *TAP-SLD5 (KanMX) pep4Δ::URA3 ADE2*	This study
YCS394	*MAT***a** *MCM4-5-FLAG (hphNT) leu2-3,112:: pRS305-GAL-DPB2-9myc (LEU2) GAL TIR1-9myc (klTRP1) dpb2-IAA17 (kanMX) pep4Δ::ADE2*	This study
YCS396	*MAT***a** *MCM4-5-FLAG (hphNT) leu2-3,112:: pRS305-GAL-dpb2-NT (1-168)-9myc GAL TIR1-9myc (klTRP1) dpb2-IAA17 (kanMX) pep4Δ::ADE2*	This study
YCS1180	*MAT***a** *MCM4-5FLAG (hphNT) pep4Δ::ADE2*	This study
YGDP1970	*MAT***a** *CTF18-TAP (HIS3MX) pep4Δ::URA3 ADE2*	This study
YGDP1971	*MAT***a** *CTF18-TAP (HIS3MX) dcc1Δ::hphNT pep4Δ::URA3 ADE2*	This study
YGDP1972	*MAT***a** *DCC1-TAP (HIS3MX) pep4Δ::URA3 ADE2*	This study
YGDP1973	*MAT***a** *DCC1-TAP (HIS3MX) ctf18Δ::K.l.TRP1 pep4Δ::URA3 ADE2*	This study
YGDP993	*MAT***a** *MCM4-5FLAG (hphNT) mrc1Δ::K.l.TRP1 pep4Δ::ADE2*	This study
YLG33	*MAT***a** *TAP-SLD5 (KanMX) ctf18Δ::K.l.TRP1 pep4Δ::URA3 ADE2*	This study
YLG60	*MAT***a** *trp1-901 leu2-3,112 ura3-52 his3-200 gal4Δ gal80Δ LYS2::GAL1-HIS3 GAL2-ADE2 met2::GAL7-lacZ ctf8Δ::URA3*	This study
YLG63	*MAT***a** *trp1-901 leu2-3,112 ura3-52 his3-200 gal4Δ gal80Δ LYS2::GAL1-HIS3 GAL2-ADE2 met2::GAL7-lacZ dcc1Δ::URA3*	This study
YLG98	*MAT***a** *MRC1-18MYC (K.l.TRP1) pep4Δ::URA3 ADE2*	This study
YLG100	*MAT***a** *MRC1-18MYC (K.l.TRP1) CTF18-TAP (HIS3MX) pep4Δ::URA3 ADE2*	This study
YLG263	*MATΔ / MATα ade2-1 / ade2-1 ura3- 1 /ura3-1 his3-1 / his3-1 trp1-1 / trp1-1 leu2-3, 112 / leu2-3,112 can1-100 / can1-100 ctf18-2A (K.l.TRP1) / CTF18 ctf4Δ::KanMX / CTF4 pep4Δ::ADE2 / PEP4*	This study
YLG249	*MAT***a** *ctf18-2A (K.l.TRP1) pep4Δ::ADE2*	This study
YLG284	*MAT***a** */ MATα ade2-1 / ade2-1 ura3- 1 /ura3-1 his3-1 / his3-1 trp1-1 / trp1-1 leu2-3, 112 / leu2-3,112 can1-100 / can1-100 CTF18 (K.l.TRP1) / CTF18 ctf4Δ::KanMX / CTF4 pep4Δ::ADE2 / PEP4*	This study
YLG292	*MAT***a** */ MATα ade2-1 / ade2-1 ura3- 1 /ura3-1 his3-1 / his3-1 trp1-1 / trp1-1 leu2-3, 112 / leu2-3,112 can1-100 / can1-100 CTF18 (K.l.TRP1) / CTF18 mrc1Δ::hphNT / MRC1 pep4Δ::ADE2 / PEP4*	This study

YLG296	*MAT***a** */ MATα ade2-1 / ade2-1 ura3- 1 /ura3-1 his3-1 / his3-1 trp1-1 / trp1-1 leu2-3, 112 / leu2-3,112 can1-100 / can1-100 ctf18-2A (K.l.TRP1) / CTF18 mrc1Δ::hphNT / MRC1 pep4Δ::ADE2 / PEP4*	This study
YLG301	*MAT***a** *DCC1-TAP (HIS3MX) CTF18 (K.l.TRP1) pep4Δ::URA3 ADE2*	This study
YLG303	*MAT***a** *DCC1-TAP (HIS3MX) ctf18-2A (K.l.TRP1) pep4Δ::URA3 ADE2*	This study
YLG316	*MAT***a** *CTF18 (K.l.TRP1)*	This study
YLG320	*MAT***a** *ctf18-2A (K.l.TRP1)*	This study
YLG421	*MAT***a** *MCM4-5-FLAG (hphNT) ctf18Δ::K.l.TRP1 pep4Δ::URA3 ADE2*	This study
YLG423	*MAT***a** *MCM4-5-FLAG (hphNT) ctf18-2A (K.l.TRP1) pep4Δ::URA3 ADE2*	This study
YLG426	*MAT***a** *MCM4-5FLAG (hphNT) CTF18 (K.l.TRP1) pep4Δ::URA3 ADE2*	This study
YLG445	*MAT***a** *CTF18 (K.l.TRP1) ura3::3xURA3-tetO112 leu2::tetR-GFP-LEU2 PDS1-mCherry (KanMX)*	This study
YLG447	*MAT***a** *ctf18-2A (K.l.TRP1) ura3::3xURA3-tetO112 leu2::tetR-GFP-LEU2 PDS1-mCherry (KanMX)*	This study
YLG449	*MAT***a** *ctf18Δ::K.l.TRP1 ura3::3xURA3-tetO112 leu2::tetR-GFP-LEU2 PDS1-mCherry (KanMX)*	This study
YVM164	*MAT***a** *ctf18Δ::K.l.TRP1*	This study
YVM657	*MAT***a***CTF18-TAP (HIS3MX) POL2-9MYC (K.l.TRP1) RFC4-6HA (K.l.TRP1) pep4Δ::URA3 ADE2*	This study
YVM738	*MAT***a** *CTF18-TAP (HIS3MX) POL2-9MYC (K.l.TRP1) RFC4-6HA (K.l.TRP1) dcc1Δ::hphNT pep4Δ::URA3 ADE2*	This study
YVM740	*MAT***a** *CTF18-TAP (HIS3MX) POL2-9MYC (K.l.TRP1) RFC4-6HA (K.l.TRP1) ctf8Δ::hphNT pep4Δ::URA3 ADE2*	This study
YVM850	*MAT***a** *DCC1-TAP (HIS3MX) ctf18Δ::K.l.TRP1 pep4Δ::URA3 ADE2*	This study
PJ69-4A	*MAT***a** *trp1-901 leu2-3,112 ura3-52 his3-200 gal4Δ gal80Δ LYS2::GAL1-HIS3 GAL2-ADE2 met2::GAL7-lacZ*	H. Ulrich

Supplementary Table S1

Strains used in this study – all the above strains are based on the W303 background, except for the yeast two-hybrid strain PJ69-4A and its derivatives YLG60 and YLG63.

Plasmid	Description	Source
pAD27	Gal4 DNA binding domain-MYC-Inn1	This study
pAD30	Gal4 Activation domain-HA-Hof1	This study
pGADT7	Gal4 Activation domain-HA	Clontech
pGBKT7	Gal4 DNA binding domain-MYC	Clontech
pHM27	pGADT7-Mrc1	This study
pLG1	pGADT7-Ctf18	This study
pLG2	pGBKT7-Ctf18	This study
pLG4	pGBKT7-Ctf8	This study
pLG6	pGBKT7-Dcc1	This study
pLG16	pGBKT7-Pol2 1-1256	This study
pLG24	pGADT7-Ctf18 1-116	This study
pLG25	pGADT7-Ctf18 1-374	This study
pLG26	pGADT7-Ctf18 1-494	This study
pLG27	pGADT7-Ctf18 1-595	This study
pLG28	pGADT7-Ctf18 1-657	This study
pLG31	pGADT7-Ctf18 369-741	This study
pLG32	pGADT7-Ctf18 494-741	This study
pLG35	pGADT7-Ctf18 654-741	This study
pLG36	pGADT7-Ctf18 675-741	This study
pLG37	pGADT7-Ctf18 709-741	This study
pLG54	pGBKT7-Pol2 1-227	This study
pLG55	pGBKT7-Pol2 1-396	This study
pLG57	pGBKT7-Pol2 1-933	This study
pLG59	pGBKT7-Pol2 214-1265	This study
pLG60	pGBKT7-Pol2 392-1265	This study
pLG61	pGBKT7-Pol2 627-1265	This study
pLG62	pGBKT7-Pol2 930-1265	This study

Supplementary Table S2

Plasmids used in this study.
